# Quantification of tumor burden in multiple myeloma by atlas-based semi-automatic segmentation of WB-DWI

**DOI:** 10.1186/s40644-020-0286-5

**Published:** 2020-01-13

**Authors:** Sílvia D. Almeida, João Santinha, Francisco P. M. Oliveira, Joana Ip, Maria Lisitskaya, João Lourenço, Aycan Uysal, Celso Matos, Cristina João, Nikolaos Papanikolaou

**Affiliations:** 1 Computational Clinical Imaging Group, Champalimaud Foundation, Centre for the Unknown, Av. Brasília, Doca de Pedrouços, 1400-038 Lisbon, Portugal; 2 Radiopharmacology, Champalimaud Centre for the Unknown, Av. Brasília, 1400-038 Lisbon, Portugal; 3 Radiology Department, Champalimaud Centre for the Unknown, Av. Brasília, 1400-038 Lisbon, Portugal; 4 Hematology Department, Champalimaud Centre for the Unknown, Av. Brasília, 1400-038 Lisbon, Portugal; 5 Immunology Department, Nova Medical School, Nova University of Lisbon, 1169-056 Lisbon, Portugal

**Keywords:** Diffusion weighted imaging, Semi-automatic segmentation, Atlas-based segmentation, Total lesion burden, Multiple myeloma

## Abstract

**Background:**

Whole-body diffusion weighted imaging (WB-DWI) has proven value to detect multiple myeloma (MM) lesions. However, the large volume of imaging data and the presence of numerous lesions makes the reading process challenging. The aim of the current study was to develop a semi-automatic lesion segmentation algorithm for WB-DWI images in MM patients and to evaluate this smart-algorithm (SA) performance by comparing it to the manual segmentations performed by radiologists.

**Methods:**

An atlas-based segmentation was developed to remove the high-signal intensity normal tissues on WB-DWI and to restrict the lesion area to the skeleton. Then, an outlier threshold-based segmentation was applied to WB-DWI images, and the segmented area’s signal intensity was compared to the average signal intensity of a low-fat muscle on T1-weighted images. This method was validated in 22 whole-body DWI images of patients diagnosed with MM. Dice similarity coefficient (DSC), sensitivity and positive predictive value (PPV) were computed to evaluate the SA performance against the gold standard (GS) and to compare with the radiologists. A non-parametric Wilcoxon test was also performed. Apparent diffusion coefficient (ADC) histogram metrics and lesion volume were extracted for the GS segmentation and for the correctly identified lesions by SA and their correlation was assessed.

**Results:**

The mean inter-radiologists DSC was 0.323 ± 0.268. The SA vs GS achieved a DSC of 0.274 ± 0.227, sensitivity of 0.764 ± 0.276 and PPV 0.217 ± 0.207. Its distribution was not significantly different from the mean DSC of inter-radiologist segmentation (*p* = 0.108, Wilcoxon test). ADC and lesion volume intraclass correlation coefficient (ICC) of the GS and of the correctly identified lesions by the SA was 0.996 for the median and 0.894 for the lesion volume (*p* < 0.001). The duration of the lesion volume segmentation by the SA was, on average, 10.22 ± 0.86 min, per patient.

**Conclusions:**

The SA provides equally reproducible segmentation results when compared to the manual segmentation of radiologists. Thus, the proposed method offers robust and efficient segmentation of MM lesions on WB-DWI. This method may aid accurate assessment of tumor burden and therefore provide insights to treatment response assessment.

## Background

Multiple myeloma is a hematologic neoplasia characterized by an abnormal proliferation of malignant plasma cells throughout the bone marrow with specific diagnosis criteria [1, 2]. Whole-body diffusion weighted imaging (WB-DWI) has a proven value to detect and follow-up of MM lesions [3]. The International Myeloma Working Group (IMWG) holds that magnetic resonance imaging (MRI) is the gold standard for the detection of bone marrow involvement in MM [4]. Whole-body MRI (WB-MRI) protocols have now been standardized, which include T1-weighted (T1w), short tau inversion recovery (STIR) and diffusion weighted imaging (DWI) [5]. Infiltrative bone marrow pathologies, such as MM, show high cellularity patterns and water content while decreasing the amount of fat. As a result, MM lesions often present with low signal on T1w spin echo MRI, being easily distinguishable from yellow marrow, while showing restricted diffusion. Apparent diffusion coefficient (ADC) is an imaging biomarker that quantifies diffusion processes within the tissues, and it is related to the ratio of intracellular and extracellular water diffusivity. It has been proposed as a potential imaging biomarker to assess treatment response. Hypercellularity or cell swelling causes contraction of the extracellular space, resulting in restricted diffusion of water molecules, as indicated by a low ADC value; on the contrary, tissues with low cellularity or necrotic areas exhibit an increase of ADC value. Combined with signal intensity on high b-value DWI images, ADC has been shown as a good indicator of the biophysical properties of bone metastases [6–9].

One of the biggest challenges for radiologists when evaluating WB-MRI images is the increased volume of data. Additionally, in patients with a large number of MM lesions and several patterns of bone marrow involvement, the quantification of the total lesion burden by means of segmentation is challenging when using manual delineation, on a slice by slice manner. Furthermore, the choice of a limited number of lesions, occasionally engaged due to time constraints, instead of the total lesion volume may lead to wrong treatment response assessment. Lesion segmentation is challenging and depend on several factors, including low image quality, a large number of lesions and limited contrast resolution. These problems may be solved by utilizing information from multiple contrasts mechanisms and by combining different segmentation algorithms (thresholding, region-growing, clustering) [10]. Although region-growing algorithms to delineate lesions are emerging [11], significant user’s interaction is still necessary to define seed points or a threshold that segments the lesion. Another big challenge, related to infiltrative bone marrow pathologies, is the inherent difficulty to distinguish between disease infiltration and hematopoietic marrow due to reconversion after treatment or hematopoietic stimuli in patients with MM [12].

In this work, we propose a novel smart algorithm (SA) that: i) removes organs presented with high signal intensity on WB-DWI (spleen, kidneys, spinal cord, bladder and testis) based on WB atlas registration, ii) restricts the lesion area to the skeleton and nearby areas, also based on a WB atlas registration and iii) segments suspicious areas on DWI images of MM patients, utilizing T1 information. The purpose of the current study was to develop a fast, semi-automatic segmentation method that is robust irrespectively of the types of MM lesion patterns and that could be used to assist radiologists in the accurate quantification of total lesion burden with implications on treatment response assessment.

## Methods

### Patients and imaging protocol

Forty WB-MRI from MM patients, acquired between 2014 and 2018, were consecutively selected by a hematologist from our MM database. The Hemato-Oncology department of our institution keeps record of all patients with MM that are diagnosed and followed in our institution. Four radiologists (3 specialists and one resident) assessed these datasets. The inclusion criteria were that WB-DWI (b value of 0 and 800 or 1000 s/mm^2^) and WB-T1w were available, that no severe anatomical deformities, distortion artifacts or implants were present and that at least one lesion was found by all radiologists (*N* = 22). Consequently, a retrospective analysis was performed on these 22 WB-MRI datasets, obtained from 16 patients (10 male age 67 ± 13 years; 6 female age 70 ± 6 years) diagnosed with MM. Two female and three male patients had multiple WB-MRI exams during treatment. From the 22 WB-MRI, at the time of the exam, four had Monoclonal Gammopathy of Unknown Significance (MGUS) and 18 had MM, from which 13 had active disease according to the IMWG criteria; two MGUS and three MM were diagnosed at that date; all the others relapsed. Images were anonymized being nominated by a prefix (WB) and a number (from 01 to 22).

WB-MRI was performed using a 1.5 T MRI scanner (Ingenia, Philips Healthcare, Best, The Netherlands). At least three sequences were acquired namely T1w, STIR and DWI (0 and 800 or 1000 s/mm^2^ b-value). T2-weighted images were also acquired in five cases.

**Fig. 1:**
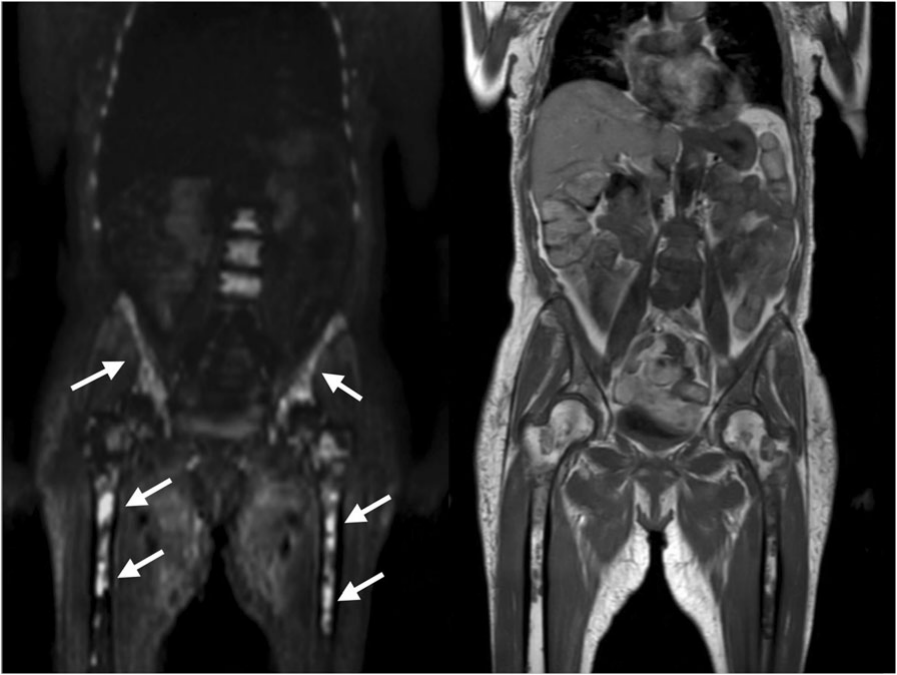
Representative coronal slice of a WB-T1w (left) and WB-DWI (right) of the same MM patient. MM focal lesions visible on left femoral head, right iliac wing and lumbar vertebral bodies, show hyperintense signal on b800/1000 image (right) and hypointense signal on T1w image (left)

Four to five axial DWI sequences were acquired in different anatomical levels covering the WB of the patient, with 44–79 slices, using free breathing single-shot echo planar imaging. Multiplanar reconstructions were made by stitching together different axial stations and reconstructing in the coronal plane, in order to generate a single WB diffusion dataset. Each DWI sequence was acquired with 2 b-values (0 and 800 or 1000 s/mm2), using the following parameters: repetition time (TR): 6219–12,075 ms; echo time (TE): 63–90 ms; acquisition matrix: 1628 × 902; slice thickness 4–7 mm; pixel spacing: 1.5–1.8 mm. For the T1w acquisition the parameters were: TR 412 ms, TE 4 ms, acquisition matrix 484 × 1219 or 484 × 980, slice thickness 5–6 mm and pixel spacing 2.09 × 2.09 or 1.14 × 1.14. An example of a reconstructed WB-DWI and WB-T1w in the coronal plane, where lesions are well visible, is shown in Fig. 1.

This dataset was used for validation of the semi-automatic segmentation method proposed.

### Semi-automatic segmentation

The semi-automatic segmentation can be divided into three main steps: removal of the hyperintense organs based on WB atlas registration [13, 14]; selection of the skeleton and nearby regions, also based on a WB atlas registration; and, finally semi-automatic segmentation on the selected regions based on threshold methods.

Since we did not have a DWI atlas, we started our work by constructing one, as described below. Once created, it was used in this study but can be applied in other similar studies.

#### DWI atlas building

Seventy-four high b-value (800 or 1000 s/mm2) DWI images from 74 patients (42 male age 69 ± 10, 25 diagnosed with MM, 11 with prostate carcinoma and 6 with follicular non-Hodgkin lymphoma or carcinoma of the transverse colon or nodular lymphoma or clear cell kidney carcinoma; 32 female age 62 ± 10, 32 diagnosed with MM) were selected and anonymized to build a representative DWI atlas. There was no overlap between this dataset and the one used to validate the SA. The difference in the b-values played no role in the construction of the algorithm. Patients gave written informed consent. This representative DWI atlas was achieved by registering (geometrical alignment) the images to each other and then computing their average.

The registration algorithm used to build the image atlas comprised the computation of an optimal rigid transformation followed by affine and a free-form transformation. Multi-resolution registration with four levels was used on both rigid and affine transformations, while one resolution was used for the free-form. The adaptive stochastic gradient descent optimization algorithm with mutual information as similarity metric was used with a maximum number of iterations of 255. Linear interpolation was used for image resampling. The rigid transformation was initialized based on the image’s geometric center. For the free-form deformation model, a grid of control points was placed over the fixed image and the deformation field was determined based on the displacements of the grid. The transformation between control points was chosen to be propagated by cubic B-spline, since they provide excellent alignments with a low computational cost [13].

**Fig. 2:**
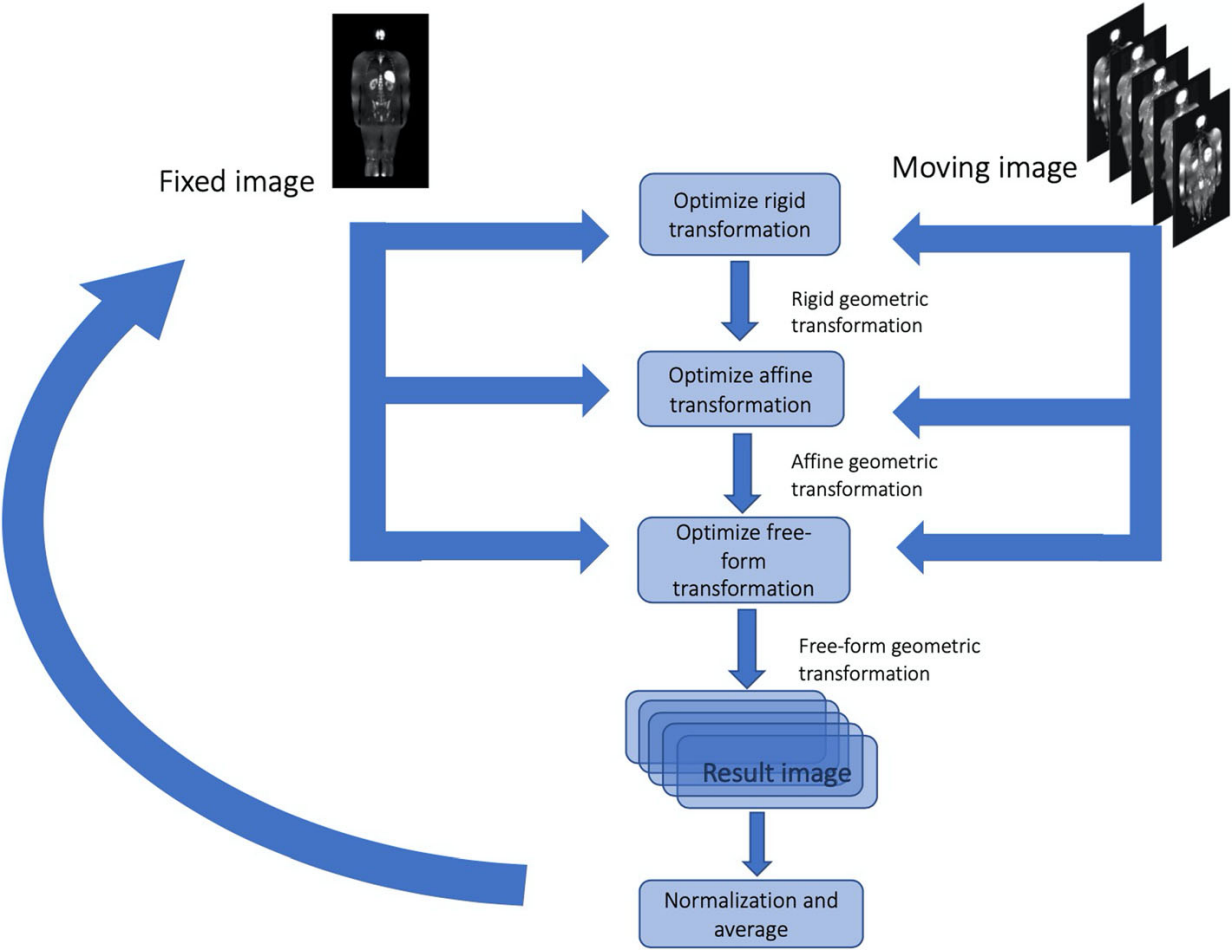
Atlas building scheme. Initially, an image is selected as the reference (fixed) and the others are moving images. Each moving image is registered to the fixed image, by applying an optimal rigid transformation, followed by an optimal affine and free-form transformations (B-spline), which maximizes the similarity between the fixed and moving images. Then, the registered moving images are averaged, resulting in a mean image. Then, this mean image is selected as the new fixed image and all the original moving images are again registered to it, following the same process. This was repeated until no meaningful changes were found between successive mean images

To initialize the gender atlas building, first a representative image was chosen and then all images were registered using that image as the first template (fixed image). Then, the mean image of the registered images was taken and then used as a fixed image for the next iteration of the template building process, and so on. This process was repeated three times, since none significant difference between the subsequent mean images was observed. This last mean image was then considered the final atlas. The techniques of registration were implemented in Python language, using Pycharm as the interpreter and using Simple Elastix [15]. All the steps of the atlas construction algorithm are shown in Fig. 2.

#### Atlas segmentation

Four radiologists (3 specialists and one last year resident) with 10, 7 and 5 years of experience manually segmented the normal hyperintense organs (spleen, kidneys, spinal cord, bladder, and testis) in the male and female atlases, using a semi-automatic tool Level Tracing Effect of 3D Slicer 4.8.1 [16]. Their segmentations were merged by union, i.e., the gold standard segmentation contains all voxels that were selected for at least one radiologist. This guarantees that all voxels of each organ were included.

MM lesions are mainly located in the skeleton or nearby areas. Exceptions are the extramedullary plasmacytomas which are less common, with an incidence of 7–18% in newly diagnosed MM and 6–20% in the course of the disease [17, 18]. Therefore, in addition to removing the normal hyperintense organs, the lesion search area was restricted to the skeleton and nearby areas. This was achieved by manually delineating, on 3D Slicer, the skeleton of the male and female atlases. The head was excluded due to neck glands and sinus liquid hyperintense tissues. The goal was not to have a perfect segmentation of bones, but rather a restricted area that surrounds them, approximately 2 mm.

At this point, there were two DWI atlases, one per each gender, their skeleton and hyperintense organs segmentations.

### Automatic selection of the lesions search regions

The atlas was registered to each high b-value validation DWI image, using the same algorithm used to build the atlas. Then, for each validation image, the transformation found was applied to the pre-segmented atlas, obtaining this way the location of the hyperintense organs and skeleton.

To guarantee that the hyperintense organs were completely removed and that the appendicular skeleton, especially the proximal and long bones, were included, these registered binary segmentations were dilated using a spherical kernel of 4 and 6 mm, respectively.

Finally, the hyperintense organs of the validation images were removed and the skeleton with nearby areas were selected for lesion search.

### Threshold and connected component segmentation

Lesion identification was based on the imaging characteristics of MM lesions. The lesions are of high signal intensity on DWI images but with equivalent or lower signal intensity than the muscle on T1w [19–22].

For DWI lesion identification, a threshold was defined based on the intensity distribution. Considering lesion intensities as outliers, optimal threshold was defined based on eq. 1,

1 $$ DWI\  lesion\ intensity\ge Q3+k\left(Q3-Q1\right) $$

where Q1 and Q3 are the first and third quartiles and k is a value that is adjusted for each patient. Generally, for outliers identification, k values of 1.5 or 3 are likely to achieve good results [23], however, based on our preliminary tests, the optimal k is around 4. This was applied to each skeleton restricted and organ removed image, created in the previous step.

**Fig. 3:**
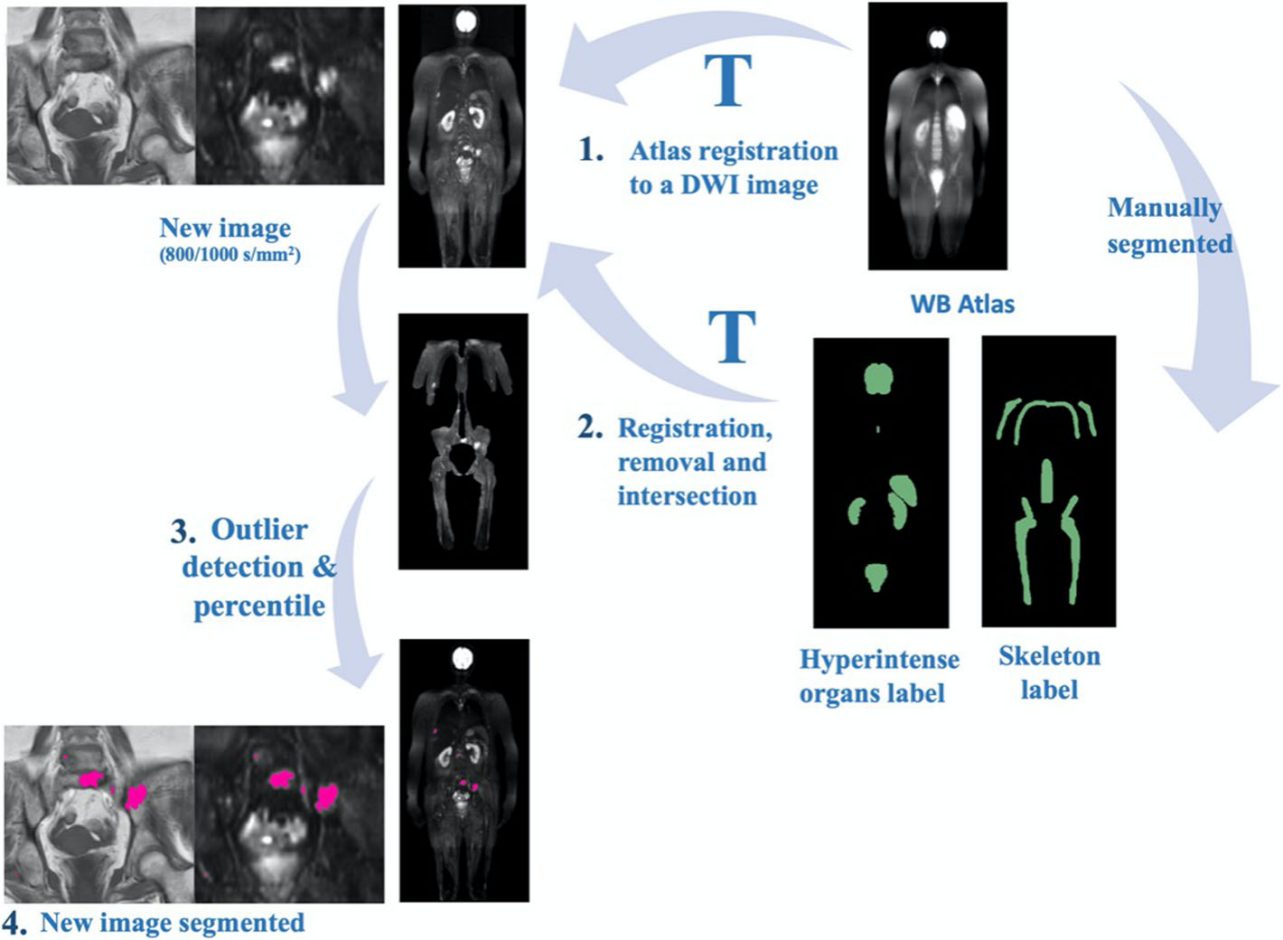
Semi-automatic lesion detection in DWI scheme. (1) The atlas is registered to a DWI image; (2) The transformation found is used to register the organs and skeleton atlas to the DWI image. Then the lesion search area is obtained by removing the organs and selecting the skeleton and nearby areas; (3) Afterwards, an automatic threshold is applied to the image, enhancing possible lesions. The T1w intensities of these possible lesions are then compared to the average of the psoas muscle to remove possible false detections; (4) The final result is shown in pink

The connected component approach grouped each threshold segmented area by connected components, to handle each lesion individually. For each patient, from the identified lesions on DWI, the ones that had a significant percentage of voxels with intensity on T1w higher than mean intensity in the psoas muscle were removed. This percentage was not fixed; it depended on the patients and on imaging quality. Based on preliminary results, the optimal percentage was around 90%. Psoas muscle was the chosen to compare lesion’s intensities since it is usually a low-fat muscle and its area extends widely in more than one slice. No size criteria were used for lesion segmentation. T1w image intensity was corrected using N4ITK MRI Bias Correction, available on 3D Slicer and registered to DWI (b0 images) using rigid transformation (Fig. 3).

### Validation

#### Manual segmentation

The manual segmentation was done by the same four radiologists that segmented the normal hyperintense organs in the atlases (E1, E2, E3, and E4) using ITK-SNAP 3.6.0 [24]. Radiologists were given access to all the WB-MRI images available (DWI, T1w, T2w, STIR) in the reconstructed coronal plane. After visual analyzing the images, they proceeded with the manual segmentation of lesions.

Radiologists’ manual segmentation of MM lesion lacks high agreement, which is supported by previous studies regarding inter-observer measurements [25, 26]. To overcome this, a majority voting method was used, where a voxel is considered as lesion if at least three out of four radiologists considered it as such. This was taken as the lesion segmentation gold standard (GS).

### Statistical analysis

Dice similarity coefficient (DSC) [14] was the metric used to assess the segmentation agreement: SA vs GS and radiologist vs radiologist. Also, sensitivity in lesion detection was assessed for SA and each radiologist in relation to the GS. A lesion was considered correctly detected if it overlapped, at least partially, with a lesion in the GS. A non-parametric Wilcoxon test was performed on IBM SPSS version 25 to compare the distributions of the DSC of the SA against GS and against the mean inter-radiologist DSC.

ADC histogram metrics (median, mean, 5th, 25th, 75th, 95th percentiles, skewness, kurtosis) and lesion volume were computed for the GS and for the correctly identified lesions by the SA (just those that have a match with the GS). Finally, the intraclass correlation coefficient (ICC) was assessed between them. ADC parametric map was calculated based on eq. 2,

2 $$ {ADC}_i=-\frac{\ln \frac{S_i}{S_0}}{b_i-{b}_0} $$

where *S*_*i*_ is the image acquired with the higher b-value (800 or 1000 s/m^2^) and *S*_0_ the lower (0 s/mm^2^). *b*_*i*_ and *b*_*o*_ are the b-values. The metrics were implemented in Python, using SimpleITK [27].

## Results

### Smart algorithm lesion segmentation

The threshold values predefined for the lesion segmentation based on the DWI and T1w images were not optimal for all patients. The k value ranged from 2.5 to 7.5, being 4 the most frequent value. The cut-off percentage to exclude the lesions based on the T1w varied between 60 and 99%, being 90% the most frequent.

The duration of the lesion segmentation by the SA was, on average, 10.22 ± 0.86 min, per patient.

**Fig. 4:**
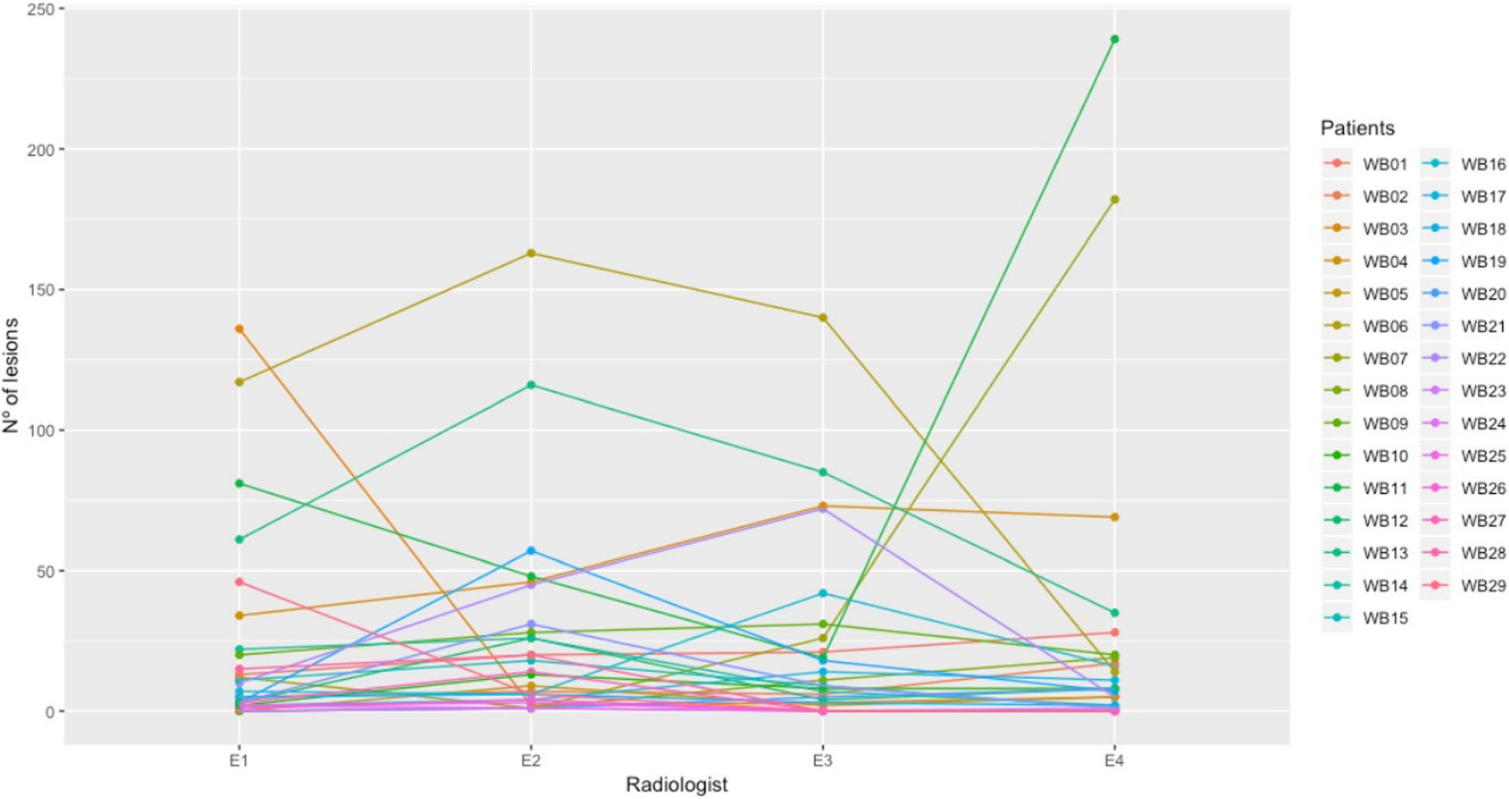
Parallel coordinate plot for the number of manually segmented lesions by each radiologist (E1, E2, E3, E4), per DWI (WB 01–22)

**Fig. 5:**
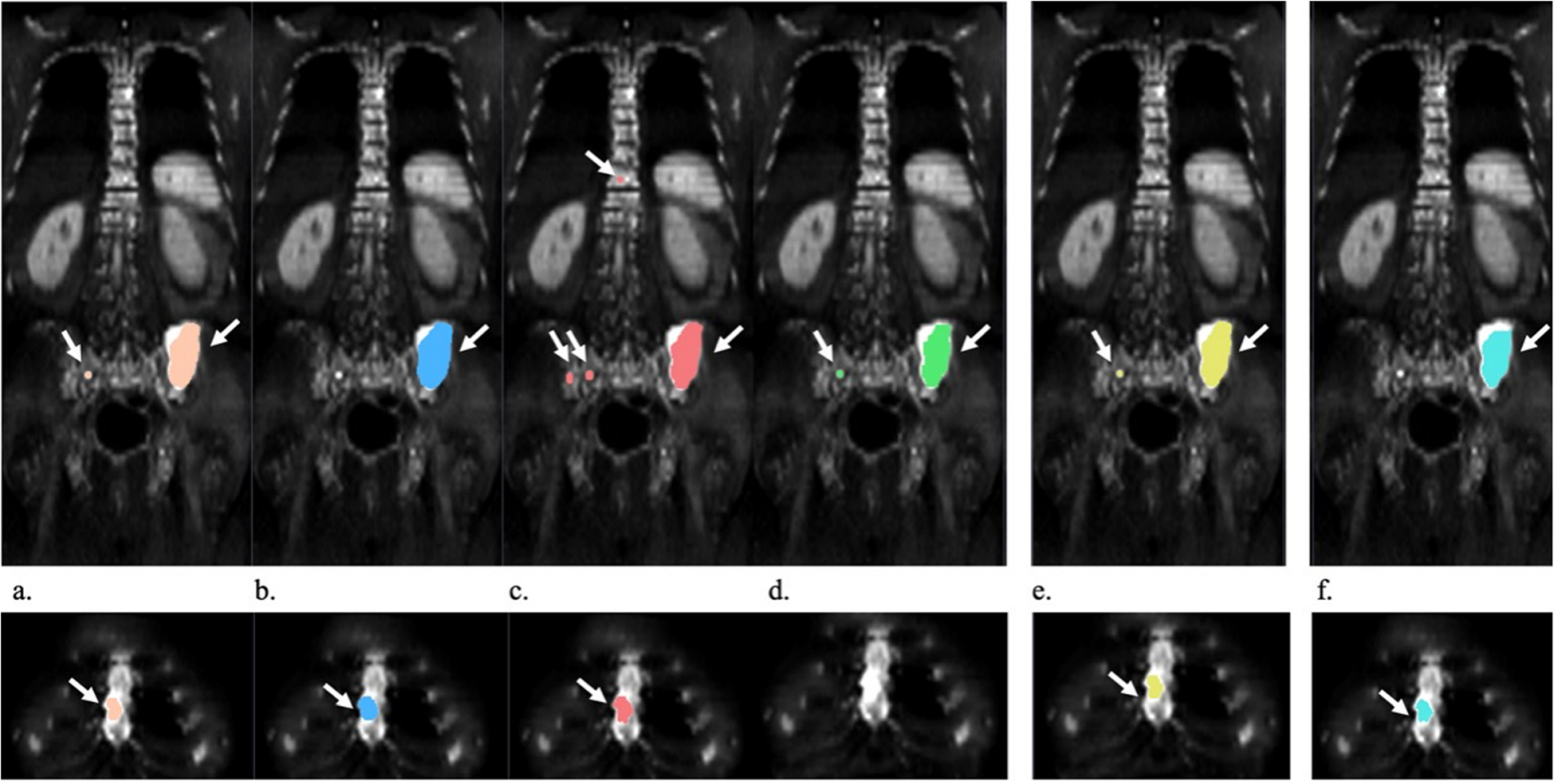
Representative coronal slices of the segmentation of the spine, pelvis and sternum. Segmentation from E1 (**a**), E2 (**b**), E3 (**c**), E4 (**d**), GS (**e**) and SA (**f**) on one image example. Segmentations are color-coded for easy reference. Identified lesions are highlighted by a white arrow. Radiologists segmented 7, 6, 42, 16 different lesions, of which 6 were identified by at least three (GS). SA identified 19 different lesions, from which 4 were correctly identified as such, which results in a sensitivity of 0.666 and PPV of 0.211. Overall, the segmentation of the lesions is consistent between the manually, GS and SA segmented images, with a greater focus on the iliac lesion

**Fig. 6:**
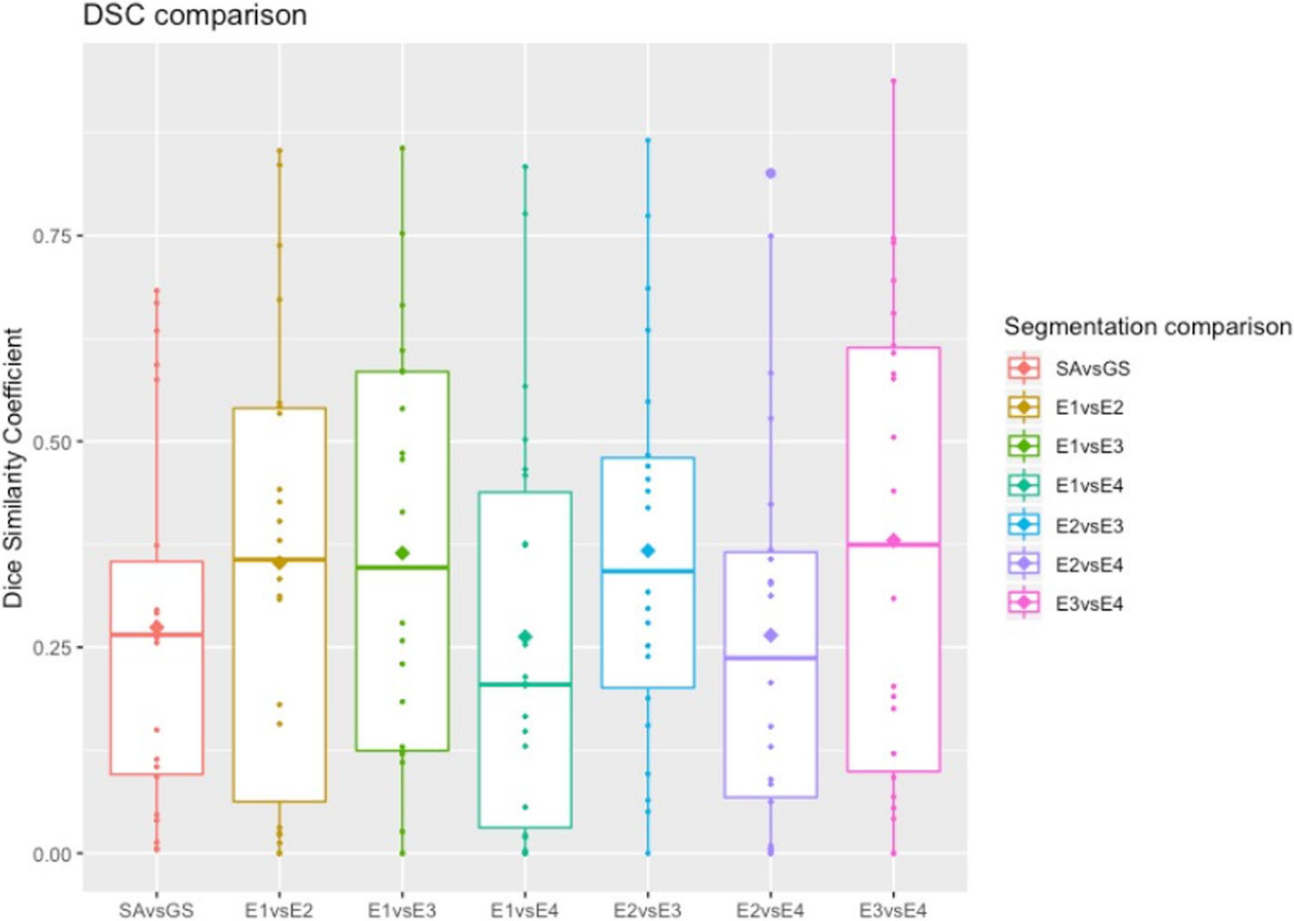
Boxplots depicting the distribution of DSC of SA vs GS and inter-radiologist. The horizontal line represents the median of the distribution while the diamond symbolizes the average

The number of lesions segmented by each of the four radiologists (E1, E2, E3, E4), per patient is presented in Fig. 4. Lesions manual segmentation done by E1, E2, E3, E4, the GS and the segmentation done by the SA on a WB image is shown in Fig. 5. According to the GS segmentation, our dataset comprised four images with 1 lesion, eight images with 2–5 lesions and 10 images with at least 6 lesions. Four images had more than 40 lesions. The mean DSC between SA vs GS and radiologists vs radiologists (inter-radiologists) are presented in Table 1. Boxplots of the distribution of DSC of SA vs GS and inter-radiologist are shown in Fig. 6.

**Table 1:** Mean DSC ± standard deviation (SD) for SA vs GS and inter-radiologist

	DSC ± SD
SA vs GS	0.274 ± 0.227
E1 vs E2	0.353 ± 0.277
E1 vs E3	0.364 ± 0.261
E1 vs E4	0.263 ± 0.252
E2 vs E3	0.367 ± 0.237
E2 vs E4	0.264 ± 0.244
E3 vs E4	0.379 ± 0.295

The DSC measured between radiologists showed a poor agreement, and therefore high segmentation variability. Considering all combinations of the four radiologists, an average DSC of 0.332 ± 0.261 was achieved. Taking into consideration that a DSC above 0.7 is suggestive of very good agreement [28], this result is much lower than would be generally acceptable. Even considering the two radiologists that showed the highest level of agreement (E3 vs E4 0.379 ± 0.295), the agreement was very low. Mean sensitivity of the radiologists was 0.836 ± 0.285.

Considering only as input the DWI images, without T1w information, the SA algorithm achieved a mean DSC against the GS of 0.229 ± 0.215, sensitivity of 0.872 ± 0.202 and PPV of 0.166 ± 0.179. Its distribution is significantly different from the mean DSC of inter-radiologist segmentation (*p* = 0.024, Wilcoxon test).

Considering as input the DWI and T1w images, the SA vs GS achieved a mean DSC of 0.274 ± 0.227, sensitivity of 0.764 ± 0.276 and PPV 0.217 ± 0.207. Its distribution is not significantly different from the mean DSC of inter-radiologist segmentation (*p* = 0.108, Wilcoxon test).

Agreement between the ADC values of the GS and that of the correctly identified lesions by the SA was excellent with ICC ranging between 0.972 and 0.996 (*p* < 0.001) for the median, mean, 5th, 25th, 75th and 95th percentile; and 0.961 for the lesion volume (p < 0.001). The ICC for the kurtosis was 0.286 (*p* < 0.079) and for the skewness 0.475 (*p* < 0.005) (Table 2).

**Table 2:** ICC and *p*-values of histogram metrics between SA and GS

Histogram metric	ICC	*p* value
Median	0.996	< 0.001
5th percentile	0.990	< 0.001
25th percentile	0.996	< 0.001
Mean	0.991	< 0.001
75th percentile	0.972	< 0.001
95th percentile	0.981	< 0.001
Volume	0.894	< 0.001
Kurtosis	0.286	< 0.079
Skewness	0.475	< 0.005

## Discussion

In this work, we developed an algorithm to improve the process of segmentation of lesions of MM in WB-DWI images, to allow accurately and rapidly quantification of total lesion burden, by validating against radiologist’s manual segmentation. Quantification of bone marrow lesions (bright on DWI) volume without considering normally hyperintense organs and other structures was made possible due to the development of an atlas-based and a smart lesion detector algorithm. The first allowed the removal of normal hyperintense organs and restriction of the search area to the skeleton or nearby areas from the images to be studied, using a suitable registration procedure. The second applied an outlier detector algorithm to delineate lesions. The segmentation performance was improved by introducing T1w information. Percentiles of single lesions obtained from the outlier segmentation were compared to the average of the intensity of psoas muscle, on T1w.

We have shown the feasibility of applying this segmentation algorithm in a group of 22 WB-DWI images, corresponding to 16 patients diagnosed with MM, in different stages of the disease and where the majority checks for the active MM criteria.

According to the revised IMWG diagnostic criteria for MM, the presence of at least two focal lesions on MRI studies with more than 5 mm in size is a biomarker of malignancy and one of myeloma defining events. According to the GS segmentation, eighteen images from our dataset had at least 2 lesions. Four images had more than 40 lesions. In normal clinical practice, total lesion volume is not assessed. Instead, the biggest lesions diameters are measured, which may be problematic due to non-spherical lesions and decision variation. If its biggest diameter is less than 5 mm, it is too small to characterize as pathological. On the other hand, might be excluding clinical findings. We opted by not to define a minimum size for lesions, assigning more freedom on this.

The high disagreement between radiologists was confirmed by the low mean DSC of the manual segmentation (0.332 ± 0.261) and the high variance in the number of lesions detected, which is shown in Fig. 4. It should be noted that, in general, the higher the number of lesions, the higher the disagreement between the radiologists (Fig. 4), which can be explained by the fatigue and complexity when having to analyze more than 60 images per sequence and counting four sequences. Furthermore, manual delineation hindered the reading process, making errors more propitious. This small test allowed to have a proof of concept about the disagreement between radiologists when defining situations with multiple lesions as in MM, which stresses the need for the development of a robust tool to assist radiologists to better and more rapidly identify lesions and to segment them. This divergence shows how difficult it is to detect and segment lesions in the common clinical practice, which results in misinterpretations between experts of the same field. Tight schedules, high workflow and lack of proper segmentation tools maybe some of the reasons for this low agreement.

The DSC of the smart-segmentation algorithm is not significantly different from the majority voting of radiologists (*p* = 0.108, Wilcoxon test), achieving a very good sensitivity (0.764 ± 0.276). The SA succeeds in identifying lesions as such but still has false positives. One cause of false positives is the presence of hyperintensities at the periphery of the field of view due to radiofrequency coil effects. When these hyperintense areas are in muscle, for instance, they may not be removed based on the T1w, and thus wrongly classified. Although the agreement SA vs GS is not the ideal, the results show that our method provides equally reproducible segmentations than the manual. Preliminary results obtained in the development stage have shown that using both DWI and T1w as inputs seem to improve the segmentation performance compared to using only the DWI images. This was expected since it is accessing further lesion information, which may help to distinguish between MM lesion and hematopoietic marrow due to reconversion.

The ICC, considering the correctly identified lesions by the SA and the GS, was 0.894 for the total lesion volume and 0.996 for the median ADC values (*p* < 0.001), which shows the reliability of the volume and median ADC measurements using the SA. This information could be used to assess treatment response by measurements of ADC and lesion volume changes between pre- and post-treatment scans and to distinguish between different MM patterns, for instance, diffuse from normal [19].

To our knowledge, there is no previous relevant work describing a semi-automatic segmentation of MM lesions on WB-DWI, using an atlas-based approach and comparing it to the segmentation of radiologists. Thus, the performance of our method cannot be directly compared to any method described in the literature. However, in a previous study, a semi-automatic segmentation model using a Markov Random Field was used to infer tumor diffusion volume and ADC, using WB-DWI [29]. Although the results were quite promising, it still requires a lot of user interaction to define the contrast between the lesion and normal tissues and to define a threshold that covers the lesions. Also, this segmentation algorithm was not validated against radiologists, since they only evaluated based on responder/non-responder to treatment. Plus, the associated computational time is of the order of 30 min. Our method benefits from the inclusion of two sequences in the analysis, which increases the confidence in the results and is faster (computational time of approximately 10 min). The latter further supports the notion that optimized semi-automatic registration methods combined with algorithms that quantitatively analyze DWI and T1w images can be used to assist radiologists while defining the total lesion burden. Also, the high ICC of the lesion volume and of the median ADC with that of GS may show the potential of extracting this global measurement out of the segmentation and use it as an indicator of response to therapy.

As far as metabolic imaging is concerned, lytic lesions and extramedullary masses present as avid for ^18^F-fluorodeoxyglucose (^18^F-FDG). Thus, positron emission tomography/computed tomography (PET/CT) exhibits high value for its identification, besides distinguishing active from inactive metabolic lesions due to the combination of functional and morphological information. According to the IMWG, ^18^F-FDG PET/CT is recommended to distinguish active from smoldering MM only when WB-MRI is unavailable and WB X-ray is negative [30]. MRI remains as the preferred and more sensitive method for the assessment of diffuse bone marrow involvement of the spine.

Among the limitations of this study is the fact that for technical reasons, some of the selected images suffered from artifactual hyperintensities that contaminated the results. There is a need for the image acquisition technique to be meticulous, thus minimizing these effects. Another limitation was the exclusion of skull and neck lesions due to technical problems in the raw images, which reduce the accuracy to quantify total lesion burden. Nonetheless, we verified that no radiologist segmented lesions above the neck. Also, the fact that the lesion area is restricted to the skeleton and nearby areas maybe excluding extramedullary plasmacytomas. However, this would not have a significant impact on the total lesion volume assessment, since the majority of lesions are found in the spine, proximal and long bones. Among the 22 WB images evaluated, 15 had previous treatment, thus T2 shine-through effect is not completely excluded just by using DWI and T1w images. Moreover, the incorporation of ADC maps or other sequences, as STIR, opposed-phase and post-contrast T1w images may increase the reliability of the segmentation helping to eliminate false positives, what would benefit this study. Another limitation is the fact that the radiologists’ reading, and segmentation time was not measured in this study. Thus, their time performance was not yet compared with the algorithm. Even though, just for a simple reference, a recent report from the Sociedad Española de Radiología Médica state that radiologists should be given 40 min to analyze WB-MRI datasets [31], without segmentation.

## Conclusions

The proposed method provides equivalent segmentations to the manual ones from radiologists on MM lesion and reproducible results on ADC histogram metrics. This study can be considered as part of the development of automatic detection and segmentation methods for MM lesions, with high sensitivity. The current smart segmentation algorithm might be useful to perform a more objective evaluation of total lesion volume and ADC quantification in order to provide a more accurate assessment of treatment response in patients with MM.

## Data Availability

The aggregated results obtained during the current study are available from the corresponding author on reasonable request.
